# Effects of zoledronic acid and dexamethasone on early phases of socket healing after tooth extraction in rats: A preliminary macroscopic and microscopic quantitative study

**DOI:** 10.4317/medoral.22883

**Published:** 2019-04-24

**Authors:** Giovanni Mergoni, Paolo Vescovi, Pietro Passerini, Roberta Maestri, Domenico Corradi, Roberto Sala, Paolo Govoni

**Affiliations:** 1 Unit of Oral Medicine and Laser Surgery; Department of Medicine and Surgery, University of Parma, Parma, Italy; 2 Unit of Pathology, Department of Medicine and Surgery, University of Parma, Parma, Italy; 3 Unit of General Pathology, Department of Medicine and Surgery, University of Parma, Parma, Italy; 4 Unit of Anatomy Histology and Embryology Department of Medicine and Surgery, University of Parma, Parma, Italy

## Abstract

**Background:**

The exact pathogenesis of medication-related osteonecrosis of the jaw (MRONJ) is still unknown. The aim of this paper was to investigate the effects of zoledronic acid and dexamethasone on the early phases of socket healing in rats subjected to tooth extractions.

**Material and Methods:**

Thirty male Sprague-Dawley rats were divided into 2 groups: pharmacologically treated group (T, n=20) and non-pharmacologically treated group (C, n=10). T group rats received 0.1 mg/Kg of zoledronic acid (ZOL) and 1 mg/Kg of dexamethasone (DEX) three times a week for 10 consecutive weeks. C group rats were infused with vehicle. After 9 weeks from the first infusion, first maxillary molars were extracted in each of the rats. Quantitative macroscopic and microscopic analysis was performed to evaluate socket healing 8 days after extraction.

**Results:**

Pharmacologically treated rats showed significant inhibition of bone remodeling. Connective tissue/alveolar bone ratio, osteoclast number and woven bone deposition were significantly reduced in group T compared to group C. Conversely, the proportion of necrotic bone was higher in group T compared to group C (0.8% and 0.3%, respectively. *P*=0.031). ZOL plus DEX do not cause gross effects on socket healing at a macroscopic level.

**Conclusions:**

Our findings confirmed that exposure to ZOL plus DEX impairs alveolar wound repair. Inhibition of osteoclastic resorption of socket walls after tooth extraction and the inability to dispose of the necrotic bone may be considered the initial steps of MRONJ onset.

** Key words:**Medication-related osteonecrosis of the jaw, zoledronic acid, dexamethasone, tooth extraction, rat.

## Introduction

Osteonecrosis of the jaw has an incidence in the general population of less than 0.001% (1). However, it increases tremendously in patients treated with high dose of antiresorptive and/or antiangiogenetic drugs, where the incidence rises to 1-15% (1). This condition, called medication-related osteonecrosis of the jaw (MRONJ), causes various jaw signs and symptoms, including pain, swelling, infection and, in some severe cases, pathologic fractures. Among MRONJ local risk factors, dento-alveolar surgery, and in particular dental extractions, are by far the most important. Indeed a rate ranging from 52-61% of patients with MRONJ reported tooth extraction as a precipitating event (2). Zoledronate (ZOL) is the antiresorptive agent most frequently associated with post extractive MRONJ (3). ZOL is a potent nitrogen-containing bisphosphonate (BP) used for the treatment of lytic lesions in patients affected by multiple myeloma and for the management of skeletal-related events associated with bone metastases. ZOL inhibits bone resorption, increasing bone mineral density and reducing fracture risks.

In some oncologic patients such as those affected by multiple myeloma, glucocorticoids are used in combination with ZOL and this association further increases the risk of post extractive MRONJ (1). Although the pathophysiology of post-extractive MRONJ is unclear, many hypotheses have been proposed, including suppression of bone turnover and an antiangiogenetic effects (4). ZOL suppresses bone turnover mainly by decreasing activity and osteoclast numbers and possesses an anti-angiogenic action by inducing capillary reduction and inhibition of endothelial vascular growth factors (5).

Several studies have investigated the effects of ZOL and dexamethasone (DEX) on extractive socket healing in rats (6-12) with observations being made 2 weeks or more after tooth extraction, but no data exists for the very early phases of the healing process.

The purpose of this experimental study is to quantitatively evaluate the effect of zoledronic acid and dexamethasone on the early phases of socket healing in rats undergone to tooth extractions.

## Material and Methods

-Animals

This study was approved by the Parma University Veterinary Sciences Committee for Ethics and by the Italian Ministry of Public Health and was planned in accordance with the guidelines for experimental procedures found in the Declaration of Helsinki of the World Medical Association.

Thirty male adult (5 week old) Sprangue-Dawley rats weighing 275 ± 25 grams were employed for the experimental procedure. Animals were housed in cages in groups of 3-4 per cage, in excellent hygienic conditions with unlimited access to food and water and maintained under artificial 12-hour day-night cycle, in a climate-controlled environment. They were acclimatized for a week before the beginning of the study.

Rats were randomly subdivided into 2 groups:

• pharmacologically treated group (T): composed of 20 rats injected with intraperitoneal zoledronic acid (0.1 mg/Kg in saline) and with intramuscular 1 mg/kg dexamethasone 3 times a week for 10 weeks; after 9 weeks from the first injection rats in this group were subjected to tooth extraction;

• non-pharmacologically treated group (C): composed of 10 rats infused with saline with the same volume and in the same way, at the same frequency and for the same duration of group T rats, and likewise subjected to tooth extraction after 9 weeks from the first injection.

The doses and time schedule of drug administration were designed according to previous studies in literature (11,13-15). The study population was asymmetrically distributed foreseeing a higher mortality rate in the T group, as observed in a previous pilot study to test the protocol and to evaluate laser therapy action on extraction socket healing in a MRONJ rat model, the results of which were not statistically significant.

-Tooth extraction

Nine weeks after the beginning of the study, rats were anesthetized with tiletamine/zolazepam (Zoletil®) and subjected to the extraction of both maxillary first molars by the same experienced physician who was blind to the experimental conditions. Surgical procedure duration (starting from the utilization of the first instrument to the end of extraction) and the incidence of tooth fracture were recorded as indicators of surgical trauma. After surgery, rats were maintained on a liquid diet for 3 days, and then returned to a standard regimen. On day 8 after surgery, all the rats were euthanized, under general anaesthesia, with an intravenous administration of 3 ml of 100 mM solution of cadmium chloride.

-Clinical assessment

At sacrifice, the maxilla of each animal was excised, surgical field was photographed using a Canon EOS 550D camera (shooting mode M, shutter speed 1/200 sec, aperture F22, 25 cm of distance from the object). Each socket was clinically assessed with the following healing grading score:

• grade 1

o socket surface wider than the occlusal area of the second maxillary molar 

o dark, rough and irregular appearance of the wound surface

• grade 2

o intermediate features between grade 1 and 3

• grade 3

o socket surface smaller than the occlusal area of the second maxillary molar

o clear, smooth and homogeneous appearance of wound surface

The final healing score for each rat was calculated as the sum of the score of right and left sockets.

The wound area of each socket was measured in pixels (pxl) with NIH ImageJ software (version 1.48). Assignment of the score and measurement of wound area were performed in a blind fashion by the same calibrated examiner.

-Histological assessment 

After clinical examination, the maxilla was immersed in a 10% buffered formalin solution and fixed for 24-48 hours. Subsequently, it was decalcified using a 10% EDTA solution (pH 7.4) for about 3 days, and transversely cut at the frontal level of extraction. The samples were embedded in paraffin tissue blocks from which 4-5 µm histological sections were obtained. Each section was then stained with haematoxylin-eosin. Quantitative histological analysis was performed by the same experienced pathologist which was unware of the assigned treatment. Images were taken under 4X-10X-40X magnification using a Nikon Eclipse 80i optical microscope, equipped with a Nikon Digital Sight DS-2Mv camera and connected to NIS Elements F control software (Nikon Instruments, Calenzano, Italy). Digital images were analysed with NIH ImageJ software (version 1.48).

The quantitative analysis of epithelium, connective tissue, alveolar bone, necrotic bone, periosteal reaction and newly-formed woven bone was carried out on haematoxylin-eosin images using the manual point counting technique as described by Vasconcelos *et al.* (16). Firstly, for each rat, the 4X magnification image of the whole socket was captured with NIS Elements F using the Grab Tool for the acquisition of large images and stored in TIFF format. Then using NIH ImageJ software a point-grid set at 1500 pixel for area per point was superimposed on each image, and each point was manually counted according to the matched morphological structure. Relative (%) values of each morphological structure were considered. Necrotic bone was defined as 8-10 adjacent empty lacunae in the alveolar bone (17). The number of osteoclasts, defined as multinucleated cells found in proximity to bone surface (18), was counted in each socket analysing images under 10X magnification. In order to evaluate vascularisation the number of blood vessels in each socket was counted in randomly chosen fields, 3 in the bone and 3 in the connective tissue, under 40X magnification. The arithmetic mean was calculated for each rat.

-Statistical analysis

Distribution of continuous data was assessed by the Skewness and Kurtosis test for normality. Normally distributed data were reported as mean and standard deviation (SD) and were compared with Student t-test for unpaired data; non-normally distributed data were reported as median and interquartile range (IQR) and were compared with Mann-Whitney U test. Categorical data were reported as frequency and percentage and compared with Fischer exact test. Count data were reported as median and IQR and compared with Poisson regression. *P* < .05 defined statistical significance. All statistical comparisons were performed with Stata version 13.0 (Stata Corp., College Station, TX).

## Results

-Clinical assessment

Animals well tolerated drug administrations, anaesthesia and tooth extractions, but 3 rats (1 rat in the C group and 2 rats in the T group) died before the end of the experiment for unknown reasons.

The elapsed time for tooth extraction, the frequency of root fractures, average wound area and the healing grading score showed no significant differences between the two groups ( Table 1).

**Table 1:**
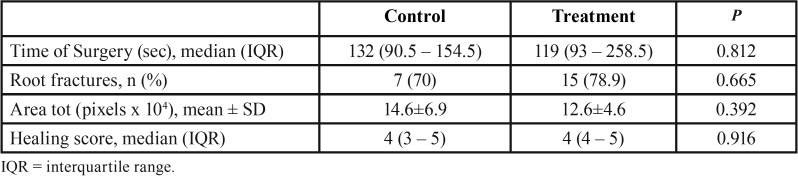
Indicators of surgical trauma and clinical assessment.

-Histologic assessment 

**Figure 1:**
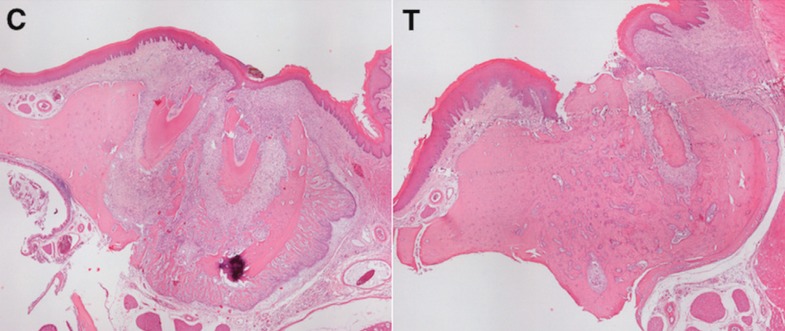
Representative histologic sections to illustrate microscopic features of socket healing 8 days after tooth extraction. Group C rats showed normal bone remodeling with extensive bone resorption of socket margins (C). Group T rats showed marked inhibition of bone remodeling with almost no bone resorption (T). (H&E, original magnification 4X).

Five specimens from the T group were excluded from the final analysis due to technical problems with histology, mainly due to the cutting phase. Representative histologic sections of socket healing 8 days after tooth extraction are shown in Figure 1 and quantitative histological analysis is summarized in  Table 2. Rats exposed to zoledronic acid plus dexamethasone showed a strong inhibition of the physiological process of bone remodeling which was observed in C group rats. Indeed, a significantly higher proportion of connective tissue was found in non-pharmacologically treated rats compared to pharmacologically treated ones (48.7 % and 24.8 %in group C and T, respectively. *P*<0.001).

**Table 2:**
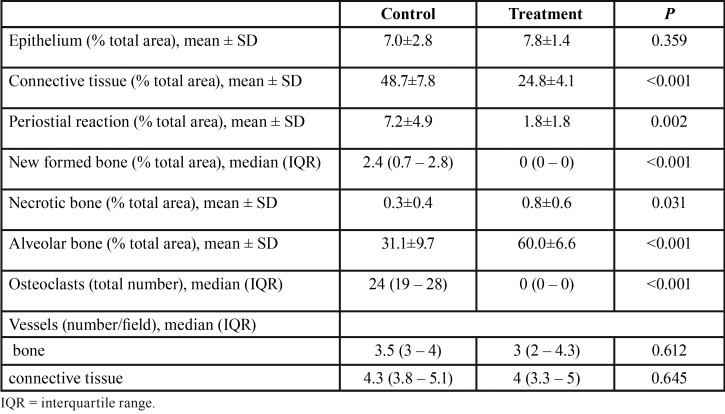
Quantitative histological analysis.

Conversely, a significantly higher proportion of alveolar bone was observed in rats from the T group compared to non-pharmacologically treated ones (60.0 % and 31.1 % in group T and C, respectively. *P*<0.001).

The osteoclast count was significantly higher in rats from group C compared to group T (*P*<0.001).

Pharmacologically treated rats showed a significant reduction in deposition of subperiosteal woven bone on the buccal surface of the socket (periosteal reaction). Similarly, the deposition of newly-formed woven bone inside the socket was reduced in group T compared to group C.

**Figure 2:**
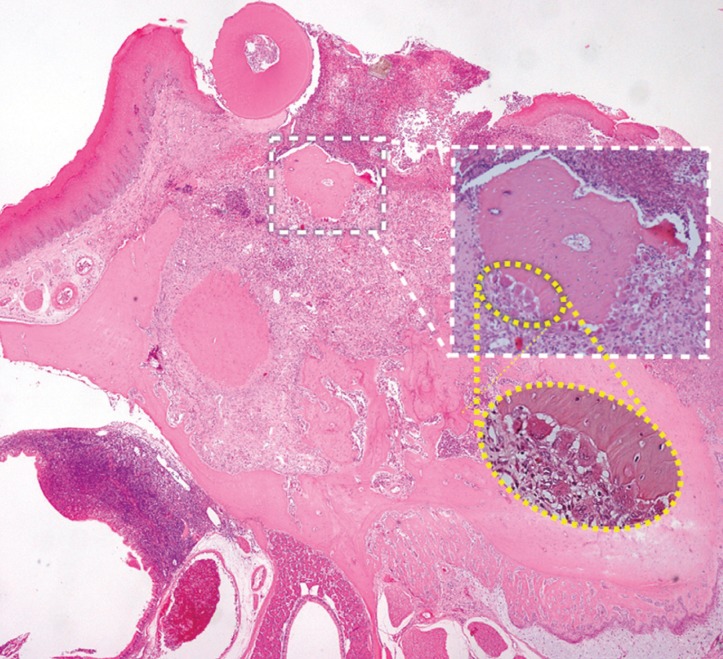
Necrotic bone actively resorbed by lining osteoclasts and expelled from the socket as bone sequestrum in a C group rat (H&E, original magnification 4X,10X and 40X).

Small areas of necrotic bone actively resorbed by osteoclasts were present in group C specimens. In some cases necrotic bone was set free from the underlying vital bone and sloughed into the socket to be expelled as bone sequestra (Fig. 2).

**Figure 3:**
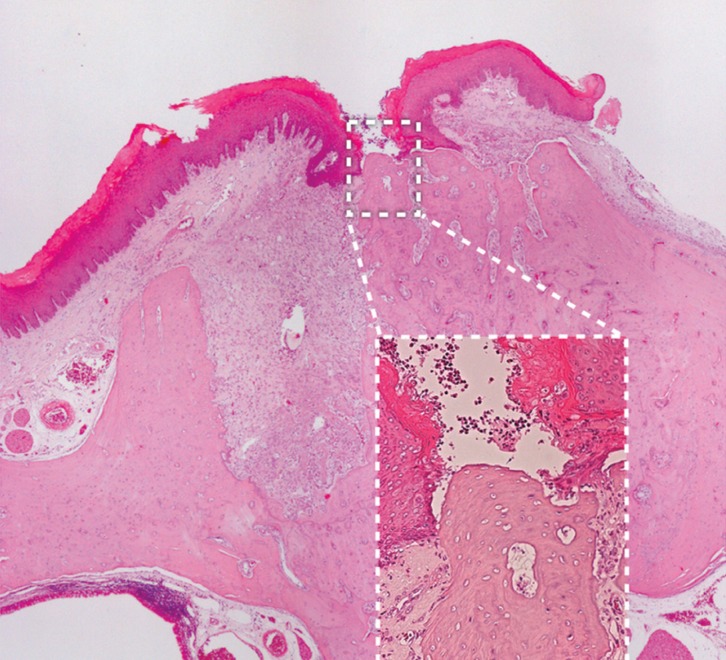
Exposed necrotic bone observed in a group T rat (H&E, original magnification 4X and X10).

Areas of necrotic bone which were not lined by osteoclasts were observed in specimens from group T and, in half of the microscopic samples, the epithelium was ulcerated with exposition of the necrotic bone (Fig. 3). The proportion of necrotic bone was significantly higher in group T compared to group C (0.8% and 0.3%, respectively. *P*=0.031)

No significant differences were found in terms of proportion of epithelium and number of blood vessels in the bone and connective tissue.

## Discussion

This experimental study confirmed the impairment effects of zoledronic acid plus dexamethasone on the early phases of socket healing after extraction in rats.

The normal healing process of the extractive socket has been extensively studied in rodents (19). In normal conditions, the blood clot is replaced by a richly-vascularized granulation tissue while intense osteoclastic bone resorption occurs at the socket margins and the alveolar crest. Necrotic bone and bone debris are resorbed in this phase. Simultaneously, osteoblast differentiation and proliferation lead to initial woven bone deposition in the apical and lateral regions. A widening of the residual alveolar ridge is often present because of the deposition of subperiosteal woven bone on the buccal maxillary surface. Bone deposition continues until connective tissue within the socket is entirely substituted by immature mineralized tissue. These features were consistently present in specimens obtained from non-pharmacologically treated rats. By contrast, pharmacologically treated rats showed a strong inhibition of osteoclastic bone resorption. Toxic effects of nitrogen-containing bisphosphonates (e.g. zoledronic acid) on osteoclasts are well documented and include changes in the cytoskeleton, loss of raffled border, disruption of actin rings and altered vesicular trafficking, leading to their inactivation and potential apoptosis (20). These effects are mainly due to inhibition of farnesyl pyrophosphate synthase, an enzyme of the mevalonate biosynthetic pathway (20). In rats exposed to zoledronic acid the osteoclast count was significantly lower compared to non-exposed rats causing a reduction in alveolar bone resorption. Indeed, the alveolar bone/connective tissue ratio was reversed when comparing pharmacologically and non-pharmacologically treated rats, with the latter showing a higher proportion of connective tissue over bone.

The above reported inhibition of bone resorption could be due not only to zoledronic acid exposure, but also to DEX. While humans exposed to glucocorticoids exhibit elevated bone resorption, the same drugs in rats cause osteoclast apoptosis (21). Byers *et al.* demonstrated a 10-fold reduction in the number of alveolar osteoclasts in rats exposed to daily dexamethasone (0.2mg/Kg) administred for 1 week (22). Hence it is likely that dexamethasone contributed to the inhibition of osteoclastic resorption observed in group T.

When alveolar bone is exposed after tooth extraction, tissues react forcefully in order to prevent osteomyelitis and osteonecrosis. This is due to the fact that exposed bone has no mechanism for dealing with inevitable bacterial colonization of the mineralized surface. Therefore, before regeneration, the exposed bone is undermined by osteoclastic resorption, leaving a soft connective-tissue surface that can defend against bacterial invasion. This was observed in rats from group C. In rats from group T zoledronate plus dexamethasone prevent undermining and disposal of necrotic bone by osteoclasts. The inability of the alveolus to dispose of necrotic bone could then lead to osteonecrosis development. Indeed, many studies have shown the onset of MRONJ-like lesions after tooth extraction in rats treated with zoledronic acid and dexamethasone (6-11,23). In our study MRONJ-like lesions were not observed, due to the insufficient observation time (8 days) after surgery which was not long enough for the development of a frank osteonecrosis.

Pharmacologically treated rats showed a reduction in deposition of newly formed woven bone at the bottom of the socket and of subperiosteal woven bone on the buccal surface of the socket (periosteal reaction) compared to controls. Osteoclasts are the main targets of bisphosphonates, but these drugs can exert biological effects on other cell populations, including osteoblasts. Basso *et al.* observed a highly cytotoxic effect on osteoblasts treated with zoledronic acid, characterized by decreased viability, total protein production and mineral nodule formation (24). Similar results were found by other authors (25). The relationship between dexamethasone and bone deposition is more complex: while it is well documented that corticosteroids can promote osteoblast differentiation (26), some studies have reported an inhibitory effect of dexamethasone on bone formation presumably mediated by glucocorticoid receptors, which have been demonstrated to be present in osteoblasts (27,28).

Inhibition of angiogenesis has been proposed as a possible mechanism of MRONJ pathophysiology (2). However, we did not observe a significant difference in terms of blood vessel numbers in the bone and in the connective tissue between groups C and T. Our findings are consistent with the study of Sonis *et al.* where no differences in vascularity were seen between controls and ZOL+DEX-treated animals (11).

We found that rats treated with zoledronic acid and dexamethasone did not show a delayed healing at macroscopic level compared to the controls. This contrasts with others studies on rat models in which gross differences of socket healing were found due to BP administration (7,8,10-12,29). A possible explanation could be that in our protocol rats were sacrificed 8 days after tooth extraction, while in the aforementioned studies macroscopic evaluation was performed after 14 days or later. It is reasonable to think that macroscopic impairing effects of medication could be more evident in the later phases of socket healing. We chose a short post-operative observation time to investigate the possible effects of ZOL plus DEX on the early phases of healing processes.

Indicators of surgical trauma (extraction time and frequency of root fractures) did not differ among the groups. According to this data it is possible to exclude differences in socket healing due to a different distribution of surgical stress between groups C and T.

The major limitation of our study was that we didn’t use immunohistochemical assays to investigate the vascolarization and the number of osteoclasts. We chose traditional and less sophisticated methods for histomorphometrical analysis because this was a preliminary study, whose results would help us to plan future experiments with more sensitive techniques. Another limitation was the lack of a group treated only with ZOL and of a group treated only with DEX in order to compare the single medication with the ZOL+DEX combination. We choose to limit the group number and, consequently, the number of sacrificed rats for ethical and economical issues.

Our study confirmed the impairing of socket healing caused by zoledronic acid and dexamethasone allowing us to detect early microscopic alterations that represent the initial steps towards MRONJ onset.

Since MRONJ is a severe condition causing a considerable burden for patients and clinicians, further in vivo research on animal models is needed to better clarify the pathogenetic mechanism of MRONJ in order to elaborate effective preventive strategies.
